# Mad River Dental Society

**Published:** 1883-01

**Authors:** 


### Mad River Dental Society.

This society met in the parlor of the Phillips House, Dayton, Ohio, Tuesday, October 24, 1882.

The following old members were present:

J. Taft, A. Berry, C. M. Wright, Cincinnati; Geo. Watt, G. L. Paine, Xenia; J. H. Paine, R. Corson, Middletown; C. Bradley, L. Hubbard, A. Sample, A. T. Whiteside, J. L. Zell, W. A. Pease, Dayton; Geo. W. Keely, Oxford; J. C. Oldham, Springfield.

New members were elected as follows:

C. I. Keely, Hamilton; J. E. Bodine, West Manchester; W. H. Sillito, Xenia; H. A. Hubbard, Charles Elson, L. C. Adams, B. A. Satterthwait, Ed. Grosvenor, R. W. Morris, Dayton.

The society has twenty-six members, all being present except one—Dr. I. N. Foote, of Middletown.

Dr. Geo. Watt read a historical sketch of the society, which he was requested to complete for publication. Old members having knowledge of interesting events connected with the old society are requested to forward accounts of same to Dr. Watt at Xenia.

Prof. C. M. Wright read a paper on Dental Prosthesis; Dr. A. Berry one on Dental Colleges and Dr. W. A. Pease one on "A New Departure" (in the treatment of alveolar abscess). A copy of these papers was requested for publication.

A committee was appointed to find the records of the society. The committee consists of Drs. Bradley and Tizzard, of Dayton, and A. Berry, of Cincinnati.

The officers elected for the ensuing year were:

President—A. Berry, Cincinnati.

Vice President—C. M. Wright.

Secretary and Treasurer--W. H. Sillito, Xenia.

Board of Censors—Geo. L. Paine, Xenia, and L. Hubbard, Dayton.

Executive Committee--J. H. Paine, Middletown; C. Bradley, Dayton, and J. Taft, Cincinnati.

The Executive reported the following subjects for the next meeting to be held in May, 1883, at Dayton;

First.—Prosthetic Dentistry, with special reference to restoration of features. Essayists, Drs. C. Bradley and A. Sample.

Second.—The restoration of crowns upon natural roots. Drs. A. T. Whiteside, C. I. Keely.

Third.—Treatment of irregularities—proper antagonisms of natural and artificial teeth. Drs. G. W. Keely, R. Corson.

Fourth.—What should be done further to protect the public from the increasing empiricism. Drs. J. Taft, C. M. Wright.

---

Iodide of Potassium a Remedy for Frontal Headache.—Dissolve sixteen grains of iodide of potassium in one ounce of water, and of this solution take one teaspoonful in one-half wineglass of water. The effect is said to be wonderful, whether attributable to the remedy or not, freedom from headache for a whole week, when before it was almost daily, generally resulting.

